# Supplementary material for: Identification of a Cardiac Glycoside Exhibiting Favorable Brain Bioavailability and Potency for Reducing Levels of the Cellular Prion Protein
Source: Int J Mol Sci. 2022 Nov 26;23(23):14823. doi: 10.3390/ijms232314823 (PMC9736291; doi:10.3390/ijms232314823)
Supplement: Supplementary file 1 [file ijms-23-14823-s001.zip › ijms-2006242-supplementary.pdf]

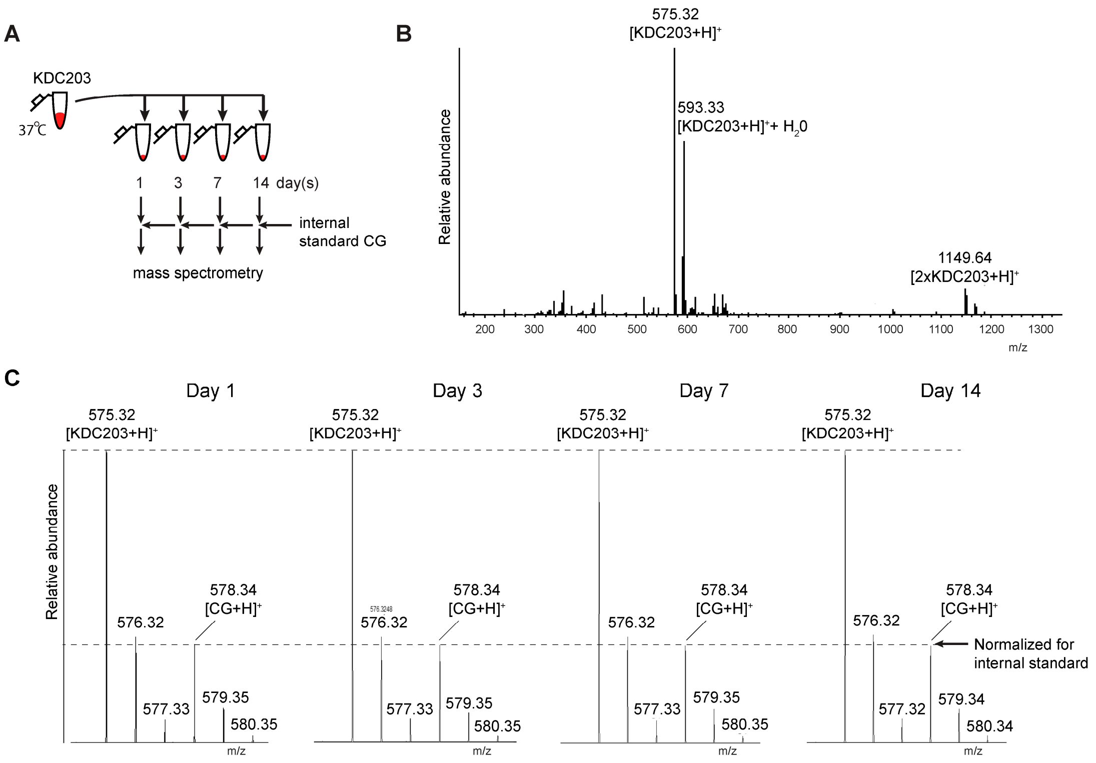

D

Days at 37°C	Intensity of monoisotopic internal standard [CG+H]+ at 578.34 Th (counts)	Intensity of monoisotopic lead compound [KDC203+H]+ at 575.32 Th (counts)	Intensity ratio of monoisotopic lead compound [KDC203+H]+ and internal standard [CG+H]+	Normalized intensity ratio of monoisotopic lead compound [KDC203+H]+ and internal standard [CG+H]+
1	3.20E+06	9.67E+06	3.02	1.00
3	3.03E+06	1.01E+07	3.33	1.10
7	5.40E+07	1.66E+08	3.07	1.02
14	3.22E+07	1.07E+08	3.32	1.10

Figure S1. Figure MS-based evidence of successful synthesis and purity of novel lead compound. (A) Design of KDC203 stability test undertaken at 36.6 °C (corresponding to the normal body temperature of mice). (B) Exemplary mass spectrum of KDC203. (C) Isotopic envelop of KDC203 with a freshly dissolved internal standard CG spiked into the sample following extended incubation but prior to sample processing for LC-MS. KDC203 continued to give rise to the base peak of the mass spectrum and no detectable degradation relative to the internal standard was detectable during the 14-day incubation. (D) Table summarizing intensities and ratios of intensities of monoisotopic peaks for KDC203 and the internal standard CG at the selected time intervals.

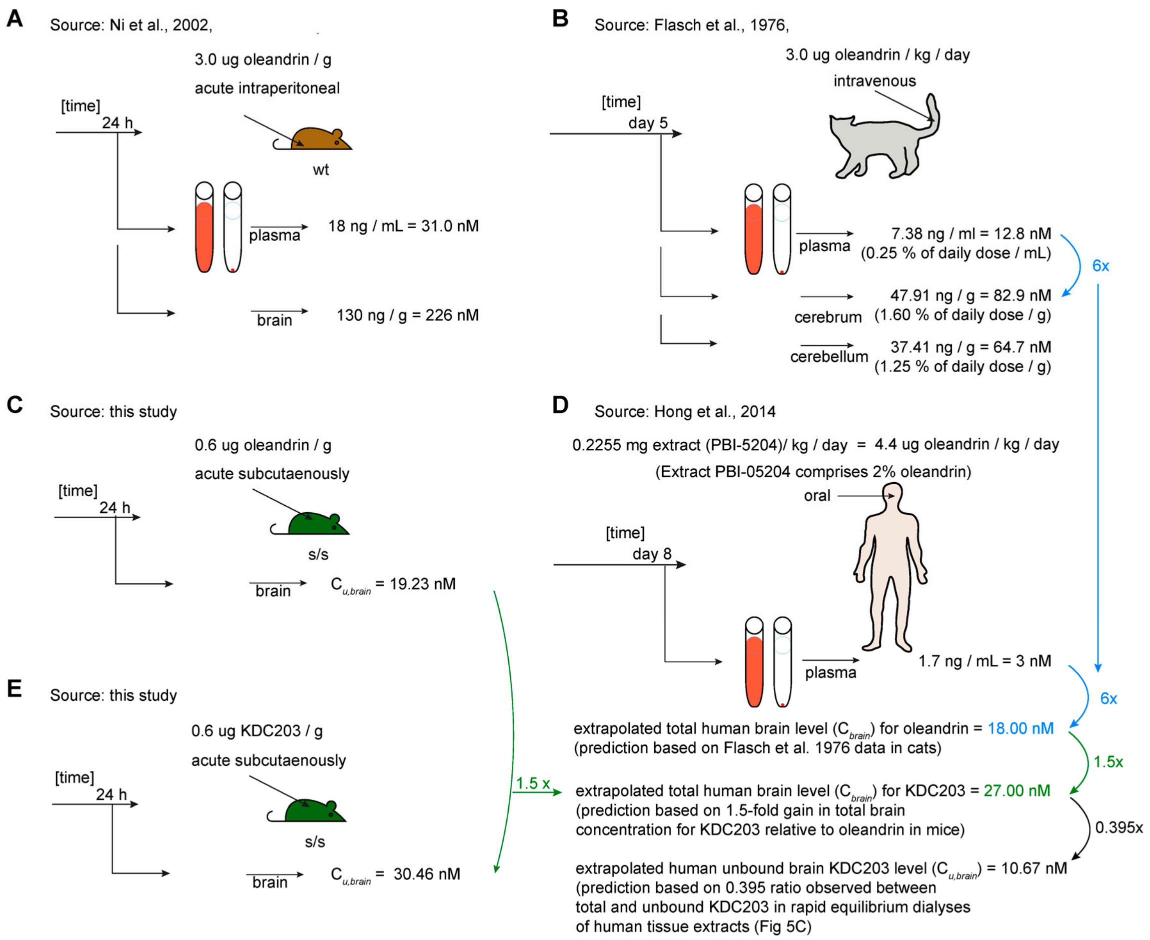

Figure S2. Figure Crude estimation of human cerebrum levels achievable with KDC203. (A) Brain and plasma levels of oleandrin achieved in wild-type mice 24 h following intraperitoneal injection of a one-time dose of 3 µg/g [31]. (B) Cerebrum and plasma levels of oleandrin observed in cats following once daily intravenous injection of 3 µg/kg [30]. (C) Average total brain concentration of oleandrin achieved in *Atp1a1*$^{S/S}$ mice 24 h following subcutaneous injection of a one-time dose of 0.6 µg/g (this study). (D) Plasma levels of oleandrin observed following daily oral administration for a duration of 8 days of 4.4 µg/kg of oleandrin present in a *Nerium oleander* extract [74]. (E) Average total brain concentration of KDC203 achieved in *Atp1a*$^{S/S}$ mice 24 h following subcutaneous injection of a one-time dose of 0.6 µg/g (this study). When estimating unbound KDC203 levels achievable in human brains, we can draw on three data points that seem most pertinent: 1. Cerebrum levels of oleandrin in the brain of cats were 6-fold higher than their plasma levels after daily administration. If a similar daily administration regime reached 3 nM oleandrin levels in the human plasma, then this might indicate that brain levels could have been as high as 18 nM. 2. Our direct comparison of oleandrin and KDC203 brain levels in mice suggests that KDC203 might reach 1.5-fold higher total brain concentrations. If this relative propensity translates to humans, then this indicates that 18 × 1.5 = 27 nM total brain concentrations of KDC203 might be attainable in human brains following an oral daily dosing regimen. 3. The RED analyses undertaken in this study with human brain samples, revealed that a ratio of 0.395 of the total KDC203 brain concentration might be available in unbound form for engagement with NKAs.
